# The effects of environmental enrichment on skin barrier recovery in humans: a randomised trial

**DOI:** 10.1038/s41598-020-66687-2

**Published:** 2020-06-17

**Authors:** Mikaela Law, Paul Jarrett, Urs M. Nater, Nadine Skoluda, Elizabeth Broadbent

**Affiliations:** 1 Department of Psychological Medicine, University of Auckland, Private Bag 92019, Auckland, 1142 New Zealand; 2 Department of Dermatology, Middlemore Hospital, Auckland, New Zealand; 3 Department of Medicine, University of Auckland, Private Bag 92019, Auckland, 1142 New Zealand; 4 Faculty of Psychology, University of Vienna, Vienna, 1010 Austria

**Keywords:** Medical research, Health care

## Abstract

This study investigated whether environmental enrichment (EE) could reduce stress and improve wound healing in humans. 120 participants underwent a standardised tape-stripping procedure and were then randomised to interact for 30 minutes with one of three EE interventions (comfort blankets as tactile enrichment, music as auditory enrichment or a Paro robot as multi-sensory enrichment) or to a control group. Skin barrier recovery (SBR) was measured using transepidermal water loss at baseline, after tape-stripping and after the intervention. Psychological variables, cortisol and alpha-amylase were measured at the three time-points. SBR did not significantly differ between the EE conditions and the control condition. The music condition had higher stimulation levels than the control condition, and the comfort condition had significantly lower relaxation levels than the control condition after the intervention. The EE interventions tested were not beneficial for wound healing compared to a control group. Limitations were that the sample were not stressed and an active control condition was used.

## Introduction

Environmental enrichment (EE) is a paradigm that involves providing sensory, cognitive and physical stimulation by altering the physical and social surroundings of the environment^1,2^. EE generally refers to the modification of a captive animal’s environment; but it has also been used to refer to enriching human environments^2^. EE has multiple categorisations that can be employed either individually or together, including: social enrichment (e.g. social support), sensory enrichment (e.g. tactile, auditory, or visual facilitation), cognitive enrichment (e.g. brain games) and motor enrichment (e.g. exercise)^2–4^.

Many studies in animals have investigated the effects of EE on health outcomes and generally found beneficial effects. For example, EE in rodents has been found to improve: cognitive functioning^5^, learning and memory^6^, immune function^7,8^, and both the behavioural^9,10^ and physiological^11–13^ stress responses. It has been proposed that EE has beneficial effects by attenuating the stress response^14^, with many studies showing that enriched animals have lower cortisol at baseline and in response to acute and chronic stress^11,15^. This reduction in stress reactivity leads to down-stream immunological and psychological benefits.

Despite substantial evidence demonstrating EE’s beneficial effects in animals, few studies have been conducted in humans^16^. There are a number of issues to consider when adapting these interventions for humans. One important issue is that the definition of EE is inconsistent with no clear operationalisation. In most rodent studies, EE refers to a large cage with opportunities for exploration and the provision of novel objects including: balls, climbing structures and running wheels^17^. However, the objects provided vary between studies, as well as the size of the cage, the duration of EE and the degree of complexity^18^.

A second issue is the variable definition of the control condition, which causes further difficulties with generalisability. In many studies, a control environment is where animals are reared in simple cages with only the necessary bedding, food and water available^19^. However, this can be seen as a state of impoverishment, with enrichment being closer to the normal state for wild animals^16^. Therefore, the provision of EE actually normalises the typical living condition and rescues the deficits caused by impoverishment. If this is true then EE may not be as effective in human populations who already live in enriched conditions.

A third issue is that animals usually have continuous access to EE over a period of weeks^16^. Such long experiments are impractical to replicate in humans because they would require isolating individuals for long periods of time. It is therefore unknown whether the effects of long-term EE in animals will translate to short-term EE in humans.

Despite these limitations, the beneficial effects in animals suggest there is merit in conducting research to examine whether EE has clinical potential for humans. Although most humans tend to already live in enriched environments, there is evidence to suggest that further enrichment can be added to human’s lives^19^. For humans, EE can include any environmental change that provides mental, physical, sensory or social stimulation^4^. Even though many human studies do not use the term EE, we can still observe EE’s benefits through studies on cognitive training, sensory enhancement (e.g. art, music, movies or video games), social support, exercise and education^3,4,19^.

There are a number of applications of EE including the provision of activities to older adults in nursing homes, such as art therapy, physical exercise, social activities and brain training^20^. Attempts have also been made to provide EE in stroke units to enrich hospitalisation^21,22^, where patients are provided with computers, reading materials, puzzles and video games. Post-stroke patients provided with EE were less likely to be inactive or asleep^21^, were more active and had fewer falls^22^, had improved depression, stress, and cognitive functioning scores^23^.

These human studies suggest that the effects of EE seen in animals may translate to humans. However, more rigorous research is still required. The current study aims to investigate how EE could improve wound healing in humans. Wound healing is an important outcome as it is clinically relevant and can be applicable for people with chronic or acute wounds. Poor wound healing can lead to infections, complications and longer hospital stays and can affect the patient’s quality of life, morbidity and mortality, as well increase healthcare costs^24^. Non-healing wounds are particularly pertinent in populations at-risk of poor healing due to lowered immune function, such as older adults, people with diabetes and obese individuals^25^.

Fricchione and Levine^26^ propose that sensory input from the environment can facilitate wound healing. The authors state that sensory deprivation can impair wound healing, whilst the provision of EE could have the opposite effect. They theorise that this link between the environment and wound healing occurs though the ‘environment-brain-skin circuit’^26^. In this circuit, sensory input from the environment is processed through the sensory cortex, which in response alters hypothalamic-pituitary-adrenal (HPA) activity and reduces the level of cortisol in the bloodstream. This is the opposite effect that stress has on HPA axis activity, and therefore EE could buffer the detrimental effects of stress on wound healing.

In support of this theory, one study has examined the effects of tactile enrichment on wound healing in rodents. Vitalo and colleagues^27^ randomised rats into three conditions: group reared rats, isolation reared rats (a stressor) and isolation reared rats provided with nest-building material (nestlets) as a form of tactile EE. The researchers investigated the rate at which the rats recovered from a burn wound. Findings indicated that the isolation reared rats provided with nestlets had significantly faster wound healing compared to the isolation reared rats without nestlets. Even more importantly, the isolation reared rats with EE and the group reared rats showed no significant differences in wound healing, indicating the nestlets reduced the negative effects of isolation on healing. Nest building behaviour is a key aspect of social bonding, and has been shown to increase maternal behaviours, and reduce stress, possibly via central nervous system activity. The effects of providing the nestlets on wound healing were similar to the effects of administering oxytocin, a social bonding hormone. However, this research is still grounded in animal models and has yet to be tested in humans.

The primary aim of the current study was to investigate whether EE could improve wound healing in humans. As the area of EE is broad with many differing types, this research employed an exploratory approach to investigate whether different forms of sensory EE could improve wound healing, including: tactile (soft blankets), auditory (music), and a combination of social, tactile and auditory (the companion robot Paro), compared to a control condition.

The tactile condition used blankets as the human version of the nestlets used in Vitalo and colleague’s study^27^. The auditory enrichment condition used music because music has been shown to decrease stress^28,29^ and improve immune parameters^30,31^. The combination enrichment condition used Paro, a companion robot designed to resemble a baby harp seal and provide comfort to users. Using sensors, Paro responds to a person’s touch and voice through movements and seal noises. Paro also has a soft fur coat which provides tactile comfort when stroked. Paro therefore represents a combination of social, tactile and auditory enrichment. Preliminary studies have shown that Paro can reduce loneliness^32^, perceived stress^33^, alpha-amylase^34^, urinary stress biomarkers^35^ and blood pressure^36^. No previous research has investigated any of these interventions on wound healing. The control condition was chosen as quiet reading, since this was the control condition used in previous wound healing research^37,38^.

The current study used a simple tape-stripping paradigm as a model of skin wound healing. This paradigm has previously been used in studies of stress and wound healing in humans, as well as an intervention study showing the provision of social support could improve healing^38^. The primary outcome for this study was wound healing via skin barrier recovery (SBR). Secondary outcomes included biological stress indices (salivary cortisol and alpha-amylase) as well as psychological variables.

It was hypothesised that the three EE conditions would show faster healing over the 30-minute recovery period, as shown by higher SBR, than the control condition. The EE conditions were also hypothesised to improve the psychological variables, and lower salivary cortisol and alpha-amylase compared to the control condition. No specific hypotheses were provided for differences between the EE conditions as no previous research has explored these forms of enrichment on human wound healing.

## Method

### Design

A 3 (time-point) × 4 (condition) mixed factorial experiment was performed to assess the effects of different forms of EE (control v comfort v music v Paro) on SBR after tape-stripping.

### Sample

A sample of 120 adults (77 female, 43 male; average age 24.64 years, *SD* = 7.98) was recruited from the community and university through flyers and email advertisements. Participants were included if they were over the age of 18 and spoke fluent English. Participants were excluded if they were allergic to tape, had an inflammatory dermatological condition (such as eczema or psoriasis), were taking medication that affected the immune system (e.g. systemic corticosteroids) or were pregnant. Ethics Approval was granted by the University of Auckland Human Participants Ethics Committee. All methods were performed in accordance with relevant guidelines and regulations.

### Power analysis

The sample size required for an adequately powered study was calculated using the programme G*Power^39^. The analysis was conducted using a linear regression model. A power level of 0.80 and alpha level of 0.05 were chosen and an expected effect size of *F*^2^ = 0.08 was estimated from a tape-stripping study on changes in SBR due to relaxation^37^. These parameters led to a required sample size of N = 103. Based on this power calculation, the 120 participants recruited meant the study was adequately powered, even with data exclusions due to issues with trans-epidermal water loss (TEWL) measurement.

### Procedure

In accordance for salivary sampling, participants were asked not to chew gum or drink caffeine, juice or alcohol 18 hours prior to the study and not to eat or brush their teeth in the hour before their session. Additionally, to prevent confounds with the TEWL measurements, they were asked not to apply moisturiser, shower or exercise in the hour before the session. Compliance with these instructions were checked at the beginning of the experimental session. All sessions were conducted between the hours of 12:30 pm and 5:00 pm to minimise the effects of diurnal rhythms on cortisol and alpha-amylase^40^. Each 90-minute session took place at the University of Auckland Clinical Research Centre. Temperature and humidity of the experimental room was measured using an ambient condition sensor RHT 100 (Courage + Khazaka, Germany).

**Figure 1:**
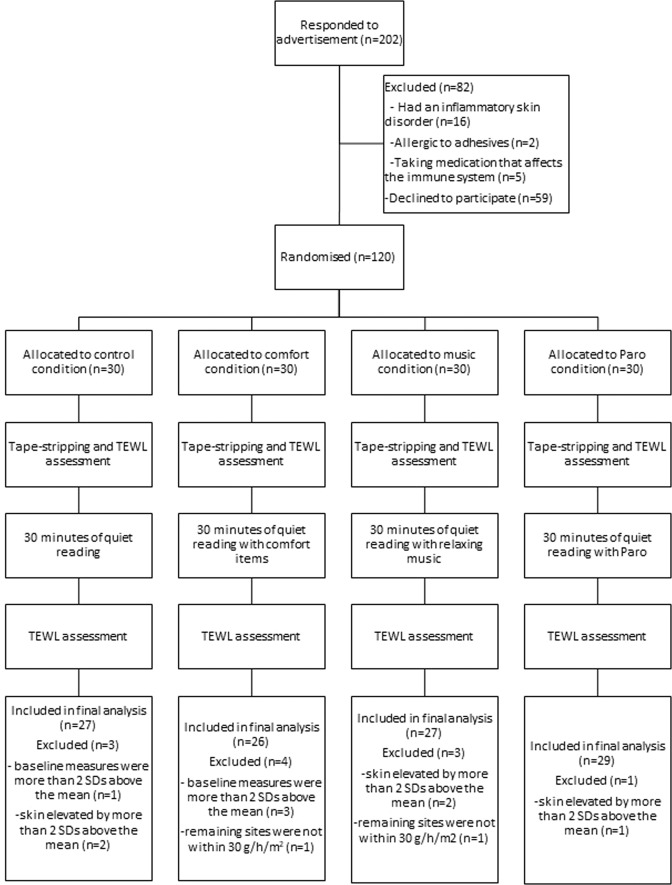
Overview of the study procedure and TEWL measurements.

The experimental procedure is shown in Fig. 1. After providing written informed consent, participants completed baseline measures including: questionnaires assessing demographics and psychological variables, a saliva sample and TEWL measures. Participants were then exposed to a standardised tape-stripping procedure to create skin barrier disruption. After this, participants completed the TEWL, psychological variables and saliva measurements again before being randomised to one of four interventions (control, comfort, music or Paro). Randomisation was performed by a researcher not involved in the experiment using a random number generator. Randomisation was concealed to the primary researcher until this point, where they opened a sealed envelope containing the group allocation.

Participants were introduced to their allocated intervention and asked to interact with their enrichment item during a 30-minute recovery period, while their experimental wound healed. A 30-minute period was chosen to investigate the immediate effect of the interventions on healing. Previous studies have successfully shown that psychological interventions can improve SBR from tape-stripping over this time-period^37,38^. Participants were asked to interact with their item as much as possible, not use any electronic devices, not touch their wound or pull down their sleeve, and not fall asleep. The researcher left the room and returned after 30 minutes. During this time, the participants were video-recorded so their compliance with instructions could be assessed. All measures were completed for a final time and participants were asked to rate how much they engaged with their item on a scale of 1 (not at all) to 5 (extremely). Upon completion of the study, participants were provided with a $40 voucher as compensation for their time.

## Interventions

### Comfort

The comfort condition was provided with tactile enrichment in the form of blankets and pillows. The participants in this condition were told to use these items throughout the 30-minute period to make themselves comfortable. This condition was seen as the human equivalent of the nestlets used in Vitalo and colleagues’^27^ study of healing in rodents. Like the nestlets, the blankets and pillows provided a sense of comfort and tactile enrichment.

### Music

Participants in the music condition were provided with a selection of CDs, a personal CD player and headphones. The participants were asked to listen to music throughout the 30 minutes. They were free to choose tracks from the CD selection provided which all contained compilations of low-arousal relaxing or classical music. These genres were chosen as they have been found to have the most reliable stress-reducing effects^28^.

### Paro

Participants in the Paro condition were provided with the robot, Paro, and asked to interact with it for the 30-minute recovery period. They were instructed that they could talk to it, stroke it and cuddle it over the recovery period.

### Control

The control condition was not provided with any form of EE other than magazines. All conditions were provided with magazines as this has been used as a control condition in previous research as a neutral activity to keep participants occupied during the 30-minute recovery period, so they did not become too bored^38,41^.

### Measures

As shown in Fig. 1, all outcome measures were taken at three time-points throughout the session; at baseline, after tape-stripping and after the 30-minute recovery period.

### Tape-stripping and SBR procedure

Tape-stripping is an experimental wound technique in which the top layer of skin, the stratum corneum, is removed by repeatedly applying and removing adhesive tape to create skin barrier disruption. Tape-stripping is a relevant model to observe wound healing in a non-invasive and inexpensive way which can also be assessed within a short time-frame. The procedure for the tape-stripping followed published methods used in previous research^37^.

Baseline skin barrier function was measured by obtaining readings of TEWL using a Tewameter TM300 probe (Courage + Khazaka, Germany), which measures the evaporation rate (in g/m^2^h) in the air layer adjacent to the skin^42^. TEWL indicates the ability of the skin to prevent water loss through the epidermis. A higher TEWL value indicates a higher amount of water is evaporating through the epidermis and lower skin barrier function. TEWL increases when the skin is damaged and returns to baseline as the skin heals, providing an estimate of SBR^42^.

To measure TEWL, four 1 cm^2^ areas were marked out on the inside of the participant’s non-dominant forearm, 1cm below the elbow crease. The bottom site was a control site which remained undisrupted. Before the baseline measures, the Tewameter probe was heated to 34 °C by a probe heater to ensure it was close to skin temperature. The participants placed their non-dominant forearm flat on a cushion and the probe was placed against each of the four marked sites, measuring each site for 60 seconds with one measurement being taken every second.

Once these baseline measures were obtained, the three test sites were dry shaved so that no hair was pulled out during the tape-stripping. The tape-stripping procedure used standard packaging tape (Scotch Commercial Grade Packaging Tape, 3 M), which was applied to the three test sites with pressure and then carefully peeled off. This was repeated 20–40 times to remove the stratum corneum. After the first 20 strips, TEWL was measured to determine whether the skin barrier had been disrupted to a minimum of 15 g/m^2^h above baseline. If the TEWL was not elevated enough, another 10 strips were applied and the skin barrier was tested again. Tape-stripping stopped when the skin barrier was elevated by at least 15 g/m^2^h or after 40 strips. Once tape-stripping had ceased, all four sites were re-measured using the Tewameter to determine the level of skin barrier impairment caused by the tape-stripping procedure.

For the rest of the experiment, participants were asked to keep their forearm uncovered and not to touch or scratch their wound. At the end of the session, TEWL was again measured to investigate SBR. A faster return to baseline indicates faster healing.

### TEWL analysis

20 consecutive measurements with a standard deviation below 0.5 were averaged to give an overall TEWL value for each site and time-point. Using these values, SBR was calculated as a percentage based on the following formula;

$$({{\rm{TEWL}}}_{{\rm{elevated}}}-{{\rm{TEWL}}}_{{\rm{recovery}}})/({{\rm{TEWL}}}_{{\rm{elevated}}}-{{\rm{TEWL}}}_{{\rm{baseline}}}){\rm{\times }}100$$

A higher percentage indicates higher SBR over time^43^. There was large variation in TEWL across the sample. This could be due to many reasons, including the fact that the humidity and temperature of the experimental room was not controlled^44^. The data was therefore screened to ensure only valid data was included. As shown in Fig. 1, SBR values were excluded if; a reading was not taken at all three time-points (n = 1), baseline TEWL reading was more than two SDs above the mean (n = 4), and skin was damaged by more than two SDs of elevation above the mean (n = 4), as these represented outliers in the data.

After this exclusion, if the SBR values of the remaining sites were all within 10 g/m^2^h all sites were averaged. Otherwise, the closest two SBR values were averaged. If the remaining sites were not within 30 g/m^2^h of each other, the values were excluded as this indicated that a reliable reading was not obtained (n = 2). After exclusion there were 27 participants in the control condition, 27 in the music condition, 26 in the comfort condition and 29 in the Paro condition.

### Salivary stress biomarkers

Saliva samples were collected at each of the three time-points as per protocol using SaliCaps collection device (IBL, Hamburg, Germany). These samples were taken to examine whether any of the interventions caused changes in stress biomarkers (cortisol and alpha-amylase). Before the first sample was taken, participants rested for 15 minutes, while completing their baseline questionnaire. Participants were asked to rinse their mouths with water, before collecting saliva using the passive drooling technique. Participants were asked to collect their naturally secreted saliva in their mouths for two minutes by not swallowing, before transferring the accumulated saliva to the SaliCap. The samples were stored at −20 °C at the University of Auckland before they were shipped on dry ice to the University of Vienna, where they were stored at −20 °C until analysis. Salivary cortisol was measured using a commercially available enzyme-linked immunosorbent assay (ELISA, IBL, Hamburg, Germany). Salivary alpha-amylase activity was determined using a kinetic colorimetric test (for details, see^45^) using reagents obtained by Roche (Roche Diagnostics, Mannheim, Germany). Intra- and inter-assay coefficients of variance of both tests were below 10%.

### Demographics and psychological measures

At baseline participants were asked about their gender, age, weight, height, alcohol consumption, smoking status, medication, diet and sleep patterns. These behaviours were assessed as they can affect immune parameters and healing.

Alcohol consumption was assessed as how often the participant had reported drinking alcohol over the past three months from 0 (never) to 5 (everyday), as well as how many drinks they had on average on the days they did drink. Participants were also asked how often they engaged in 30 minutes or more of physical activity during an average week from 0 (never) to 7 (everyday). They were also asked to rate their diet over the past week from 1 (very poor) to 5 (very good). Lastly, participants were asked, over the past month, how many hours of sleep they usually had per night to assess sleep duration.

### Perceived stress

The 10 item Perceived Stress Scale (PSS)^46^ was measured at baseline to evaluate the extent to which participants viewed their life as being stressful. Participants were asked how much they had felt a certain way over the last month on a scale of 0 (never) to 4 (very often). Scores were totalled to give a perceived stress score ranging from 0 to 40.

### Psychological variables

Visual analogue scales were completed at each time-point to assess participants’ current levels of stress, pain, anxiety, relaxation and stimulation. Participants were asked to mark a cross on a 100 mm line to represent how they were currently feeling. The anchors on the visual analogue scales included: not at all stressed to extremely stressed; no sensation of pain to most sensation of pain imaginable; not at all anxious to very anxious; not at all relaxed to very relaxed; and very bored to very stimulated.

### Statistical analysis

Data was analysed using IBM SPSS Statistics 22. A 2-step hierarchical regression was conducted to analyse the effects of condition on SBR, controlling for known covariates in the first step of the model. A series of 2-step hierarchical regressions were also conducted to analyse the effects of condition on the post-recovery levels of the salivary stress hormones and psychological variables, controlling for baseline levels of the outcomes in the first step of the models. Due to the categorical nature of the conditions, condition was dummy coded before being added to the analysis so that the three EE conditions (comfort, music and Paro) were compared to the control condition. The cortisol and alpha-amylase data violated the assumption of normality and therefore were transformed using a natural log transformation and logged values were used.

## Results

### Sample characteristics

The baseline and demographic characteristics for the sample are shown in Table 1. A summary of the statistics of the secondary outcomes across the time-points and conditions is provided in Table 2.

**Table 1:** Summary of Demographic and Baseline Characteristics of Participants across Condition.

Baseline variable	Control	Comfort	Music	Paro
Age (years) M(SD)	22.47(4.07)	24.00(6.45)	27.30(12.04)	24.80(6.73)
Gender, n(%)				
Female	20(67%)	18(60%)	21(70%)	18(60%)
Male	10(33%)	12(40%)	9(30%)	12(40%)
Ethnicity, n(%)				
NZ European	7(23%)	8(27%)	9(30%)	6(20%)
Chinese	7(23%)	10(33%)	5(17%)	8(27%)
Other	16(53%)	12(40%)	16(53%)	16(53%)
BMI M(SD)	23.11(3.93)	23.24(3.68)	22.18(5.00)	22.99(3.08)
Exercise (days/week), M(SD)	3.57(2.06)	3.90(2.19)	3.67(1.90)	4.17(1.80)
Sleep Duration (hours/night), M(SD)	7.27(0.91)	6.87(0.90)	7.10(0.89)	7.13(1.00)
Perceived Stress Scale, M(SE)	13.60(1.49)	14.20(1.24)	15.93(1.06)	14.57(1.24)
Baseline TEWL, averaged across the disrupted sites (g/h/m^2^), M(SD)	16.45(3.80)	17.34(7.24)	16.72(3.33)	16.36(3.44)
Room temperature (°C), M(SD)	25.71(1.00)	25.91(1.35)	25.78(1.14)	25.72(1.10)
Room humidity (%), M(SD)	46.52(6.68)	46.84(6.87)	46.31(6.08)	46.01(6.42)
Average level of skin barrier impairment across the disrupted sites(g/h/m^2^), M(SD)	18.92(8.28)	21.41(7.87)	17.31(8.13	19.06(9.33)
Number of strips used, n(%)				
20	10(33%)	8(27%)	11(37%)	10(33%)
30	9(30%)	15(50%)	11(37%)	9(30%)
40	11(37%)	7(23%)	8(27%)	11(37%)

*Note:* M = Mean, SD = Standard deviation, TEWL-transepidermal water loss, % = percentage of participants in that category.

**Table 2:** Summary Statistics of the Secondary Outcome across Conditions at each Time-point.

Variable	Condition	Baseline, M(SD)	Post tape-stripping, M(SD)	Post recovery period, M(SD)
VAS stress score	Control	40.67(27.32)	30.17(25.41)	16.70(19.66)
	Comfort	39.47(29.84)	26.73(25.88)	17.87(20.58)
	Music	40.83(26.00)	25.53(21.39)	13.47(13.86)
	Paro	41.33(26.96)	29.43(27.81)	19.93(23.04)
	Total	40.58(27.23)	27.97(24.98)	16.99(19.47)
VAS pain score	Control	5.33(11.06)	8.47(11.48)	2.20(3.43)
	Comfort	8.07(14.99)	14.80(14.90	6.57(10.26)
	Music	9.60(13.51)	12.17(12.75)	8.23(13.12)
	Paro	2.70(7.45)	8.73(6.60)	3.50(5.43)
	Total	6.43(12.23)	11.04(11.97)	5.13(9.14)
VAS anxiety score	Control	20.90(25.92)	16.83(22.72)	8.80(12.78)
	Comfort	19.40(21.17)	15.97(18.87)	12.23(16.19)
	Music	15.63(19.91)	13.37(17.19)	8.97(13.67)
	Paro	18.60(25.26)	11.73(16.78)	8.33(14.74)
	Total	18.63(22.99)	14.48(18.91)	9.58(14.30)
VAS relaxation score	Control	68.10(29.04)	76.03(23.58)	84.80(13.73)
	Comfort	68.53(23.06)	72.93(19.54)	76.77(21.10)
	Music	73.60(18.61)	77.47(15.00)	82.33(13.89)
	Paro	79.60(15.34)	75.27(25.22)	80.83(16.81)
	Total	72.46(22.33)	75.43(21.00)	81.18(16.701)
VAS stimulation score	Control	52.67(17.30)	42.47(22.75)	42.23(21.34)
	Comfort	53.83(16.91)	41.73(22.81)	47.30(20.99)
	Music	57.53(22.19)	49.30(23.29)	63.37(22.30)
	Paro	49.13(19.84)	43.87(23.31)	47.60(19.89)
	Total	53.29(19.17)	44.34(22.94)	50.13(22.35)
Salivary cortisol (nmol/l)	Control	1.71(1.11)	1.80(1.72)	1.22(1.07)
	Comfort	2.22(1.57)	1.82(1.60)	1.12(0.72)
	Music	2.08(1.24)	2.03(2.11)	1.55(0.81)
	Paro	3.12(3.31)	2.12(1.67)	1.19(0.87)
	Total	2.28(2.06)	1.94(1.76)	1.27(0.88)
Salivary alpha-amylase (U/ml)	Control	33.39(28.10)	33.35(30.36)	38.82(36.22)
	Comfort	44.36(40.22)	37.22(33.24)	38.82(36.22)
	Music	43.03(35.46)	38.30(27.55)	44.39(43.03)
	Paro	46.36(41.71)	44.19(40.41)	44.88(27.95)
	Total	41.79(36.64)	38.27(33.05)	42.99(54.30)

*Note:* M = mean, SD = standard deviation, VAS = visual analogue scale.

### SBR

**Figure 2:**
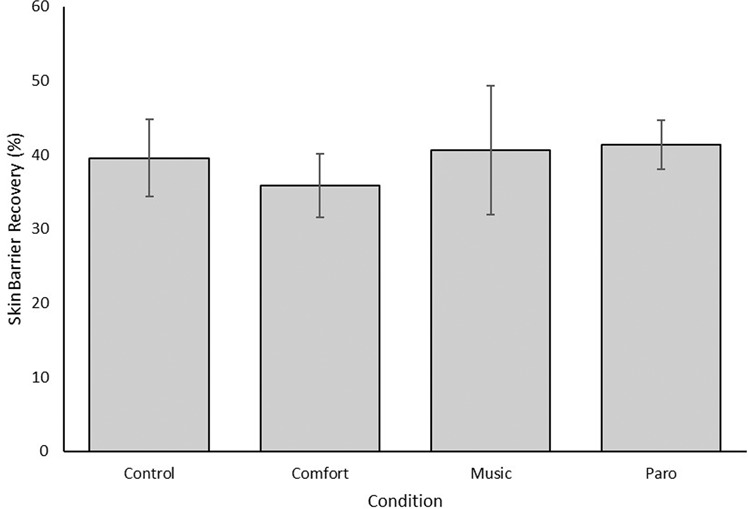
Mean skin barrier recovery across condition. Error bars represent 95% confidence intervals.

Mean SBR across conditions is shown in Fig. 2. A 2-step hierarchical regression was conducted to analyse the effects of the dummy codes for condition on SBR. The coefficients and F change scores for Step 2 of the model are shown in Table 3. Step 1 of the regression model (containing the known covariates of age, room temperature, room humidity, number of strips used and level of skin barrier impairment) was significant, indicating that these covariates explained 47% of the variance in SBR. Adding the dummy codes for condition into Step 2 of the model did not significantly explain any additional variance in the model. As shown in Table 3, none of the dummy codes were significant predictors of SBR. This indicates that SBR was not significantly different between the EE conditions and the control condition.

**Table 3:** Regression Analysis Summaries for Step 2 in the Regression Models of Dummy Codes for Condition Predicting the Outcome Measures.

Outcome	Predictor	*B*	*p*	95% CI	*ΔF*	*p*
**SBR**	(Constant)	58.48	0.078	[−6.67, 123.64]	0.20	0.895
	Comfort dummy code	1.24	0.733	[−5.94, 8.42]		
	Music dummy code	1.78	0.625	[−5.42, 8.97]		
	Paro dummy code	2.70	0.446	[−4.30, 9.70]		
**Anxiety**	(Constant)	0.33	0.879	[−4.02, 4.69]	0.85	0.468
	Comfort dummy code	4.04	0.156	[−1.56, 9.65]		
	Music dummy code	2.30	0.420	[−3.32, 7.92]		
	Paro dummy code	0.46	0.870	[−5.14, 6.07]		
**Stress**	(Constant)	−3.28	0.313	[−9.70, 3.14]	1.09	0.354
	Comfort dummy code	1.76	0.633	[−5.51, 9.02]		
	Music dummy code	−3.32	0.368	[−10.58, 3.95]		
	Paro dummy code	2.91	0.430	[−4.36, 10.17]		
**Relaxation**	(Constant)	66.84	<0.001*	[56.32, 77.37]	1.61	0.190
	Comfort dummy code	−8.15	0.046*	[−16.16, 0.13]		
	Music dummy code	−3.92	0.337	[−11.96, 4.13]		
	Paro dummy code	−7.00	0.092	[−15.15, 1.15]		
**Pain**	(Constant)	−0.97	0.334	[−2.94, 1.01]	2.54	0.060
	Comfort dummy code	2.74	0.049*	[0.01, 5.48]		
	Music dummy code	3.50	0.013*	[0.75, 6.25]		
	Paro dummy code	1.38	0.040*	[0.13, 5.60]		
**Stimulation**	(Constant)	17.15	0.005*	[5.25, 29.05]	5.22	0.002*
	Comfort dummy code	4.51	0.364	[−5.29, 14.31]		
	Music dummy code	18.82	<0.001*	[8.98, 28.65]		
	Paro dummy code	7.05	0.158	[−2.77, 16.87]		
**lnCortisol**	(Constant)	−0.26	0.005*	[−0.44, −0.08]	4.70	0.004*
	Comfort dummy code	−0.10	0.409	[−0.35, 0.14]		
	Music dummy code	0.23	0.067	[−0.02, 0.49]		
	Paro dummy code	−0.22	0.086	[−0.44, 0.70]		
**lnAlpha-amylase**	(Constant)	0.91	<0.001*	[0.44, 1.37]	1.45	0.232
	Comfort dummy code	−0.05	0.775	[−0.36, 0.27]		
	Music dummy code	0.06	0.709	[−0.26, 0.38]		
	Paro dummy code	−0.25	0.119	[−0.57, 0.88]		

*Note:* *p < 0.05, 95% CI = 95% confidence interval’s for *B*.

### Psychological variables

A series of 2 step hierarchical regressions were conducted to analyse the effects of the dummy coded condition variables on the post-recovery levels of anxiety, stress, relaxation, pain and stimulation, controlling for the effects of baseline levels of the psychological variables in step 1 of the models. The coefficients and F change scores for step 2 of the models are shown in Table 3. Step 1 of the regression models for anxiety and stress was significant. However, as shown in Table 3, the F change score for step 2 and the dummy codes for condition were not significant. This indicates that the condition the participants were assigned to did not affect the participants’ levels of anxiety or stress after the recovery period.

As shown in Table 3, the F change score for step 2 of the regression model for post-recovery levels of relaxation was not significant. However, the dummy code for comfort vs. control was a significant predictor for relaxation levels at the post-recovery timepoint. The comfort condition had a lower relaxation score than the control condition by 8.16 points, when controlling for baseline relaxation. The other dummy codes for condition were not significant predictors in the model.

As shown in Table 3, the F change score for step 2 of the regression model for post-recovery levels of pain was not significant. However, all three dummy codes were significant predictors for pain post-recovery. Compared to the control condition, the comfort condition had a higher pain score by 2.74 points; the music condition had a higher pain score by 3.50 points, and the Paro condition had a higher pain score by 1.38 points, when controlling for baseline pain values.

As shown in Table 3, the F change score for step 2 of the regression model for the post-recovery levels of stimulation was significant, indicating that the regression model for stimulation was significantly improved by adding condition as a factor. The music vs. control dummy code was a significant predictor for stimulation levels post-recovery. The music condition had a 18.82 point higher stimulation score than the control condition at the post-recovery timepoint when controlling for baseline stimulation levels. The other dummy codes for condition were not significant predictors.

### Salivary stress biomarkers

Two hierarchical regressions were conducted to analyse the effects of the dummy coded condition variables on naturally logged levels of salivary cortisol and alpha-amylase after the recovery period, controlling for the effects of baseline salivary levels of the stress biomarker in the first step of the models. The coefficients and F change scores for these models are shown in Table 3. The F change score for the cortisol model was significant, indicating that adding condition did improve the model. However, in both models, none of the individual dummy codes for condition were significant predictors. Therefore, adding condition did improve the cortisol model, but there was not enough evidence to conclude that any of the individual EE conditions improved cortisol at the post-recovery period compared to the control condition. The F change score for the alpha-amylase model was not significant, indicating that alpha-amylase levels were not affected by interaction with the EE compared to the control group.

## Discussion

This study aimed to investigate whether three types of EE (comfort items, music or a Paro robot) could improve SBR after an experimental tape-stripping wound in a healthy population. Findings indicated no significant effects of these interventions on SBR compared to a control condition. However, analysis of the secondary outcomes demonstrated that the music condition had significantly higher stimulation levels and the comfort condition had significantly lower relaxation levels at the post-recovery timepoint compared to the control condition, when controlling for baseline levels. Lastly, the results indicated that all three EE conditions had significantly higher pain levels than the control condition. However, these pain scores were still relatively low, with no conditions having pain scores over 9/100 at the post-recovery time-point.

The finding that the music condition had higher stimulation levels after the recovery period compared to the control condition may be due to distraction. Previous studies suggest that music has beneficial effects because it can distract people from negative experiences^47^. Therefore, in this study, music may have alleviated the boredom of the 30-minute recovery period.

The finding that the comfort condition had lower relaxation levels than the control condition is unexpected, as it would be expected that the addition of blankets and pillows would increase relaxation. Some participants may have been unsure how best to use these items as instructed, which may have impacted their relaxation levels.

The null finding for SBR does not support Vitalo and colleagues^27^ study, which found that EE improved wound healing in stressed rats. One of the main theories for the beneficial effects of EE on healing is via stress reduction^14,26^. Research also shows that psychological interventions, such as EE, are likely to only have an effect on immune parameters in stressed samples^48^. For example, Morley-Fletcher and colleagues^13^ found that EE only lowered the cortisol levels of rats that were chronically stressed. In non-stressed rats, no effect of EE was observed.

Therefore, one reason for the null effects found in this study for SBR may be because the sample were not sufficiently stressed and therefore may benefit less from EE compared to a possible sample experiencing stress. The sample only had low to medium perceived stress levels at baseline, and the salivary cortisol levels were relatively low throughout the study. Future studies could investigate the effects of EE on stress reduction and wound healing in a more highly stressed sample or stress the sample experimentally. However, it may also be the case that these effects seen in animals, may not be readily transferred to human population.

Another reason for the limited effects found in this study could be because previous research on EE used non-active control groups (e.g. Paro compared to no Paro, or music compared to no music); however, the current study used an active control condition. All conditions, including the control condition, had access to magazines during the recovery period. Magazines are a common control condition in research^38,41^, as they provide a neutral activity; participants don’t become too excited or enriched, but also don’t become too bored which could lead to negative impacts from lack of stimulation such as an increase in negative affect. However, the magazines could have provided some cognitive enrichment and therefore had similar effects to the other conditions.

Research on EE has shown that sensory enrichment items that provide tactile comfort (such as the nestlets in Vitalo and colleagues’^27^ research and the equivalent blankets and Paro robot in the current study) or relaxation (as shown by research on the effects of music on stress^28,29^) are key factors in how EE may improve wound healing through changes in immunological parameters and stress biomarkers. Cognitive enrichment can affect cognitive processes, such as memory and learning, but has little effect on immunological outcomes or wound healing. Therefore, the cognitive enrichment from the magazines may work through different mechanisms to sensory enrichment.

This study has several limitations. Firstly, the sample consisted of healthy participants, which makes it difficult to generalise the effects to clinical populations who are at risk of complications from poor wound healing^25^. Future research could also investigate these effects in participants with inflammatory skin disorders, who have impaired skin barrier function and investigate further skin-specific outcomes. Furthermore, the experimental tape-stripping wound may not directly represent a real-life wound. Tape-stripping causes a minor skin wound which heals over a short time-frame. This makes it difficult to directly apply these findings to clinical wounds which have different healing processes and heal over a longer period. However, a tape-stripping wound still offers some insight into how interventions may affect wound healing.

Lastly, the temperature and humidity of the experimental room varied between participants. Temperature and humidity can directly affect wound healing^49^ and TEWL readings^44^. Therefore, fluctuations in temperature and humidity may cause variation in the TEWL readings and possible inaccuracies in the measurements. Future research using tape-stripping should use a climate-controlled room where temperature and humidity are kept constant.

In conclusion, this study did not demonstrate that any of the EE interventions could improve SBR, or self-reported stress after an experimental tape-stripping wound compared to a control condition, although music did increase stimulation levels. Therefore, the effects of EE seen in animal research on wound healing may not be directly transferable to human populations. Despite these null results, the current study provides important knowledge on the potential effects of EE interventions in humans, informing future studies that will closely examine the mechanisms underlying EE interventions in stressed populations.
